# Supplementary material for: A sustainable working life in the car manufacturing industry: The role of psychosocial factors, gender and occupation
Source: PLoS One. 2020 May 14;15(5):e0233009. doi: 10.1371/journal.pone.0233009 (PMC7224489; doi:10.1371/journal.pone.0233009)
Supplement: S1 Data — (DOCX) [file pone.0233009.s001.docx]

**Supporting information**

**Occupational history was assessed by the questions:**

When did you start working at Volvo Car (Volvo Personvagnar, AB Volvo)?

När började du arbeta inom Volvo Car (Volvo Personvagnar, AB Volvo)?

What department at Volvo did you last work at/are you working at now?

Ange vilken avdelning inom Volvo du arbetade på senast / arbetar på nu

och vilken befattning har eller hade du?

At what departments within Volvo have you previously worked, and what position did you have?

På vilka avdelningar inom Volvo har du tidigare arbetat, och på vilka befattningar?

Write your previous jobs (outside Volvo) that you have had for longer than five years during your working life. Include self-employment.

Ange dina arbeten (anställningar) andra än de på Volvo som du haft längre än fem år under

ditt yrkesliv. Med anställning innefattas även arbete som egen företagare.

**Shift work was assessed by the questions:**

Have you been working shifts? (include both 2-shifts and 3-shifts)

If yes, between what years?

Har du arbetat skift? (inkluderat både 2-skift och 3-skiftsarbete)

Om Ja, mellan vilka år?

**Work hours was assessed by the questions:**

Have you been working permanent evening?

Har du arbetat ständig kväll?

Have you been working nights (not shift-work) during longer periods?

Har du arbetat nattetid (ej skiftarbete) under längre perioder?

How many hours are you working (if you are retired, how many hours did you use to work) during a typical working week?

Hur många timmar arbetar du (om du gått i pension, hur många timmar arbetade du) under en typisk arbetsvecka?
