# Supplementary material for: A sustainable working life in the car manufacturing industry: The role of psychosocial factors, gender and occupation
Source: PLoS One. 2020 May 14;15(5):e0233009. doi: 10.1371/journal.pone.0233009 (PMC7224489; doi:10.1371/journal.pone.0233009)
Supplement: Attachment — Submitted filename: Response to Reviewers.docx [file pone.0233009.s002.docx]

**Response to reviewers**

We would like to thank the reviewers and the editor for the constructive and helpful comments. We have made the following changes to the manuscript.

Note: Page numbers refer to the pages on the revised manuscript with track changes.

**Editors comments:**

Comment 1. A document has been included with supporting information including survey questions that have not been published previously.

In the methods section on page 7 citations for scales published previously have been included.

Comment 2. Updated competing interest statement. Thank you for updating the submission form on our behalf.

Volvo Cars provided contact details, age, details of level of employment and time of employment, of all employees that had worked/ and or retired at Volvo between the years of 2005-2015 that were 55 years or older. Volvo did not have any further involvement in conducting the study or in the decision to publish the study.

Comment 3. The funding statement has been updated.

The study was funded by AgeCap – a centre for aging and health at Gothenburg University and by the Swedish Research Council for Health, Working life and Wellfare. The funders did not have any involvement in the study, the writing of the manuscript or the decision to submit the paper for publication. There was no additional external funding received for this study.

Comment 4. The full name of the ethics committee has been included in the methods section on page 6, and in the submission form.

Comment 5. Data available on request.

a) Data cannot be shared publicly because information about health and symptoms are regarded at sensitive information, and when sharing such data there has to be an approval from a Swedish Ethical committee (according to Swedish law). However, anonymised data is available with an approval from an ethical review board. For data requests, contact: Department of occupational and environmental medicine, Gothenburg University, Box 414, 405 30, Gothenburg, Sweden.   The name of the data set is ‘The Volvo work ability study’.

Comment 6. Table 1 and 4 is now referred to in the text in the results section on page 10 and 14.

**Reviewer 1**

Comment 1. We agree that mentioned analyses may add more information to the decision of retiring. Exploratory analyses were conducted testing interaction effects between the main psychosocial variables (job demand-control / effort-reward imbalance) and the suggested variables (previous diseases, social support at work). The interactive effects were mostly small and all were non-significant. We conclude that these variables are not moderators, and will not add the results to our paper.

Comment 2. Exploratory analyses showed interactive effects between high strain and blue-/white-collar worker. However, the paper already includes analyses stratified by blue- white-collar worker, and illustrates effects of psychosocial variables may differ depending on being blue- or white-collar workers.

Comment 3. Table 1 is now mentioned in the text on page 10.

Comment 4. P-values are included in table 2-4 in the manuscript on pages 12, 13, 14. Some calculation errors were noted and corrected. Our results remain largely similar

Comment 5. Conclusions regarding causality has been removed on the following pages.

In the abstract on page 2.

In the aims on page 5.

In the implications on page 21.

**Reviewer 2**

Comment 1. In limitations on page 20 a sentence has been added that acknowledges that only subjective measures of working conditions were used in the study.

Casual language has been removed in the following sections:

In the abstract on page 2.

In the aims on page 5.

In the implications on page 21.

Comment 2. We evaluated several confounders we identified as important, based on previous literature and meetings with Volvo staff (including mangers, white-collar and blue-collar workers). The following variables were considered: civil status, age of partner in relation to the participant, country of birth, retirement during a year of recession, being offered a beneficial retirement deal, if retirement was the persons own decision, previous diseases, work ability at the time of retirement, social support at work, need for recovery after work at the time of retirement and stress at home at the time of retirement. Since working with a fairly small cohort, we performed stepwise purposeful selection according to Lemeshow & Hosmer (2000) reference 26, to slim our models, which resulted in the confounders presented in the paper.

We had access to other work condition variables (e.g. noise, physical demands), but our main aim with this paper was to focus on psychosocial variables. Also, this paper examines both white- and blue-collar Volvo workers. Noise and physical demands likely have little effect among white-collar workers. We plan to examine effects from physical work factors among blue-collar workers in future studies.

As suggested we examined other psychosocial variables that might be important such as managerial/executive position and overtime per week. Both variables had little effect and did not full-fill the criteria according to Lemeshow & Hosmer (2000).

We did not have access to wage level, which is a limitation, but Volvo workers at this time, where provided with a beneficial pension deal, so that pension would be similar regarding if retiring at 62 or statutory Swedish pension age 65. Wage may therefore be of less importance in this cohort, than other similar studies.

Comment 3. The limited statistical power realting to the the small sample of women is addressed in the limitations on page 20.

**Reviewer 3**

Comment 1. The reviewer highlighted that people over 55 years that were laid off appeared to be included in the sample. Individuals who had been laid off were not included in the sample. Thank you for highlighting that this was unclear. A paragraph clarifying this has been included in the study population section on page 6.

Comment 2. We agree that a major limitation of this study is the risk of recall bias. In the manuscript we have revised the text relating to limitations on page 20 to further acknowledge this problem.

Comment 3. The reviewer highlighted that the time period that the participants were asked to consider their psychosocial environment was unclear. The cases were asked to consider their current working environment, or the working environment at their last workplace at Volvo. The controls were asked to consider the working environment at their last workplace at Volvo. Information about this has been added in the section describing the questionnaire on page 7.

Comment 4. We agree that sample of women is quite small and the problem with power has been acknowledged in the section on limitations on page 20. However, despite the small sample we still think there is a value with including women in the analysis.

Comment 5. A recognition of the fact that late retirement can be problematic for certain employees have been added in the beginning of the introduction on page 3.

Comment 6. There is currently an active debate, especially regarding job demand-control, that these models that were developed several decades ago, and due to a changing labor market, may have different meaning compared to when they were created. During the time of the forming of the models, the typical high strain work situation was found in blue-collar workers e.g. in line production. Many, however, argue that due to technology, several white-collar workers in modern labor market have increasing demands linked to less boundaries and longer work hours. The understanding how to interpret and measure psychosocial work conditions needs to be updated, but to do so, there is a need to first establish limitations in standard methods.
