# Supplementary material for: A sustainable working life in the car manufacturing industry: The role of psychosocial factors, gender and occupation
Source: PLoS One. 2020 May 14;15(5):e0233009. doi: 10.1371/journal.pone.0233009 (PMC7224489; doi:10.1371/journal.pone.0233009)
Supplement: Attachment — Submitted filename: response to reviewers.docx [file pone.0233009.s003.docx]

**Response to reviewers**

**Reviewer #1**

1 & 2. We agree to these well-thought of and insightful comments, but after thorough discussion in our project group we have concluded the following. It’s correct that several of the chosen covariates could possible also be moderators. We do, however, believe that there are many studies in this field with a similar design examining psychosocial variables and using models with covariates that could be both moderators or covariates. Even though it may add interesting information, is not common to calculate or display results on interaction effects. We still carried out exploratory analyses evaluating interaction, but none were significant. In the previous review we had other models, but as suggested from reviewer 2 we included more work related covariates and interaction effects between for example psychosocial variables and socioeconomic status are now not significant anymore. We agree to your suggestion that it may benefit the study in some ways, but we have decided not to expand our results with interaction analyses, since most studies do not and since interaction effects were non-significant it may not contribute that much.

3. Thank you for this helpful suggestion. We have added a sentence about the fact that prospective studies are needed in order to find out more about casual factors. Page 19.

4. Thank you, p-value has been added to Table 4. Page 14.

**Reviewer #2**

*Contribution of the paper*

We acknowledge that this study cannot discuss causes of retirement. However, we believe that this study adds to existing knowledge regarding the associations between psychosocial factors and the timing of retirement. There is little research investigating these factors in the car manufacturing industry. We have revised the aim in order to emphasize that we are investigating the relationships between psychosocial work factors and retirement, and thereby aim to add to existing knowledge. Page 5.

*Problems with recall bias*

We agree that this can be a serious problem, and have added further reasoning regarding this issue in case-control studies in the discussion. Page 17-18.

*Problems with confounding*

We agree that our models needed to be revised and more covariates should be considered. Since modelling and used covariate is one of the main issues in the reviewer comments we have expanded the methodology section in which we explain the methodology for inclusion of covariates: stepwise purposeful selection as proposed by Hosmer & Lemeshow [1] (page 8). The rationale for using this method is that theoretically a broad variety of available covariates could be important in analyses in psychosocial work environment and retirement. However, since our cohort size is fairly small, and include analyses that are stratified by gender or blue-/white-collar workers, we wish to slim the models by only including covariates relevant for our analyses. Meaning, if a variable does not fulfill the criteria in the established selection process, they have little relevance for the model and evaluated effects.

The following potential confounders, which previous have been identified in the literature or by practical experience as important in the retirement process, were tested with stepwise purposeful selection as proposed by Hosmer & Lemeshow [1]:

civil status, age of partner in relation to the participant, country of birth, retirement during a year of recession, being offered a beneficial retirement deal, if retirement was the persons own decision, amount of years since retirement, previous diseases, work ability at the time of retirement, need for recovery after work, life events, stress at home, physical job index, quality of leadership, managerial position and social support at work.

Per suggestion we especially added work related covariates such as frequency of physically heavy job tasks [2], leadership quality [3] and leadership position in the company (yes/no). The reviewer suggest salary as an important co-variate, unfortunately we do not have access to this variable. It has been suggested that for those with high status jobs, the salary per se is not determining for retirement, but rather social status and identity tied to the position. For blue-collar workers physical health and actual retirement benefits, is more important. If health if failing, salary may have less important. Furthermore, this cohort is slightly unique as the employees, due to a very generous deal with Volvo Cars, could retire at 62 years, and receive similar pension as if they would have retired at 65 years, which was the statutory retirement age during our observation period. Thus, salary hopefully, have less importance for in this particular cohort. The reviewer also suggested we remove health as a covariate given its endogenous property in relation to work conditions. We did include it in our purposeful stepwise confounding testing, and as it now, including all new work related variables, the variable previous diseases, did not fulfil the inclusion criteria, it is no longer included in our models.

1.	Lemeshow S, Hosmer D: **Applied Logistic Regression (Wiley Series in Probability and Statistics**. Hoboken: Wiley-Interscience; 2 Sub edition; 2000.

2.	Arbetsmiljöverket: **Arbetsmiljön 2013**. In: *Arbetsmiljöstatistisk Rapport 2014:3.* vol. 2014:3; 2014.

3.	Kristensen TS, Hannerz H, Høgh A, Borg V: **The Copenhagen Psychosocial Questionnaire-a tool for the assessment and improvement of the psychosocial work environment**. *Scandinavian journal of work, environment & health* 2005:438-449.
