# Supplementary material for: A sustainable working life in the car manufacturing industry: The role of psychosocial factors, gender and occupation
Source: PLoS One. 2020 May 14;15(5):e0233009. doi: 10.1371/journal.pone.0233009 (PMC7224489; doi:10.1371/journal.pone.0233009)
Supplement: Attachment — Submitted filename: response to reviewers.docx [file pone.0233009.s004.docx]

Reviewer #1

Concern 1 & 2We deeply regret that we expressed ourselves unclear, but in the previously response letter we reported that the interaction effects between psychosocial variables and socioeconomic status (division: blue-/white-collar workers) were NOT significant anymore when entering the additional confounders requested in the previous revision by reviewer #2. So therefore, we prefer not to report these in the article. Also, if reporting on interactive effects for: psychosocial variables*other job variables; psychosocial variables * socioeconomic, then there is little justification to why we should not report interaction between main psychosocial variables and all covariates, and this would shift the focus of the study somewhat. As our study design focuses on psychosocial variables and relationships to continued work, we are not sure if this add much value, especially as the interactions mentioned are non-significant. Regarding adding the interaction because this is a justification for doing separate analyses of blue- and white-collar workers, we would argue that it is common practice to do separate analyses for these two groups. Moreover, it in the case of Volvo, it is clear that they have very different working environments.

We do, nevertheless, appreciate the reviewer’s valuable suggestions regarding how to investigate the complexity in a retirement process as multiple variables have indeed a joint effect on an executed retirement or not. We are also very interested in such joint effects on psychosocial variables and socioeconomic markers, as similar topics have arisen in other of our research projects. We therefore plan for our next study to look at retirement in clusters of persons with specific set of several physical and psychosocial work conditions and socioeconomic markers (more than just blue-/white-collar worker, which may be too simplified). However, since the design used in the current paper is in accordance with general practice in analyses with job demand-control and effort reward imbalance (two of the authors main research focus is these psychosocial job variables) we felt it important to start with a first study using a standard design, and then build on those findings to make a more complex study in our next paper.

Regarding the comment on Table 4, that we neglected to report that high ERI is associated with extended work in all, and males - we did report this in the Results, but agree that the text was unclear. We have revised the text on page 14 to make it more clear.

We have also added this information in Abstract – p 2, and in the Discussion – p 16.

Regarding the mistake in line 281, we have corrected this mistake both in the Results – line 281, and in the Discussion - page 16. Once again, we are thankful for the thorough review.
