# Supplementary material for: A sustainable working life in the car manufacturing industry: The role of psychosocial factors, gender and occupation
Source: PLoS One. 2020 May 14;15(5):e0233009. doi: 10.1371/journal.pone.0233009 (PMC7224489; doi:10.1371/journal.pone.0233009)
Supplement: Attachment — Submitted filename: Response to reviewers.docx [file pone.0233009.s005.docx]

**Response to reviewers**

**Comment from editor**: I noticed that the raw data supporting the study results is not already available in your submission. In this regard, please consider that [PLOS Data policy](http://www.plosone.org/static/policies.action#sharing) is quite strict in this regard **("PLOS journals require authors to make all data necessary to replicate their study’s findings publicly available without restriction at the time of publication. When specific legal or ethical restrictions prohibit public sharing of a data set, authors must indicate how others may obtain access to the data")**, and it must be fully available in order to accept the paper for publication.

In our response to reviewers submitted in October 2019, we included the following text. The statement includes information regarding how the data can be obtained. We hope that this is sufficient information regarding availability.

“Data cannot be shared publicly because information about health and symptoms are regarded as sensitive information, and when sharing such data there has to be an approval from a Swedish Ethical committee (according to Swedish law). However, anonymised data is available with an approval from an ethical review board. For data requests, contact: Kristina Gyllensten, Department of occupational and environmental medicine, Gothenburg University, Box 414, 405 30, Gothenburg, Sweden. The name of the data set is ‘The Volvo Work Ability Study’.

**Reviewer #1**: They do note in the Results and early in the Discussion that the finding of high strain (and ERI) association with more likelihood to continue work becomes nonsignificant in the fully adjusted models. This casts doubt on the validity of this association -- suggesting that the extensive discussion of it (lines 328-349, P. 17) should be omitted.

Thank you for pointing this out. Lines 328-349 has now been deleted.
